# Serum SP70 is a sensitive predictor of chemotherapy response in patients with advanced nonsmall cell lung cancer

**DOI:** 10.1002/cam4.1555

**Published:** 2018-05-16

**Authors:** Jingping Liu, Wei Zhang, Min Gu, Yazhou Ji, Lu Yang, Xiangjun Cheng, Xuelian Xiao, Jian Xu, Chunrong Gu, Jiexin Zhang, Shichang Zhang, Dan Chen, Shiyang Pan

**Affiliations:** ^1^ Department of Laboratory Medicine The First Affiliated Hospital of Nanjing Medical University Nanjing China; ^2^ National Key Clinical Department of Laboratory Medicine Jiangsu Province Hospital Nanjing Medical University Nanjing China

**Keywords:** clinical efficacy, nonsmall cell lung cancer, progression‐free survival, SP70, tumor markers

## Abstract

SP70 is a novel tumor biomarker in patients with nonsmall cell lung cancer (NSCLC). However, its role as a marker for predicting the response to chemotherapy for patients with advanced NSCLC has not been investigated. A total of 152 patients were enrolled. Serum SP70, carcinoembryonic antigen (CEA), cytokeratin 19 fragment (CYFRA21‐1), and neuron‐specific enolase (NSE) were detected before and after 2 cycles of chemotherapy. The correlation between serum tumor biomarker levels and chemotherapy responses and their association with epidermal growth factor receptor (*EGFR*) mutation status and progression‐free survival (PFS) were analyzed. Serum SP70 levels were significantly decreased after chemotherapy in the partial remission (PR) group (*P *<* *.001) and increased in the progressive disease (PD) group (*P *<* *.001), but not significantly changed in the stable disease (SD) group (*P *=* *.114). Although similar changes were observed on CEA and CYFRA21‐1 levels but not NSE, ROC analysis demonstrated that SP70 is superior to the others. Additionally, patients with *EGFR* mutation had higher serum SP70 levels and tissue SP70 expression than patients without *EGFR* mutation (*P *=* *.014 and *P *=* *.002, respectively). The median PFS of patients with decreased SP70 levels after chemotherapy was longer than that of patients with stable or increased serum SP70 level (24 months vs 12 months vs 2 months, *P *<* *.001), and the differences of all other 3 tumor markers were not obvious. Serum SP70 is a sensitive and real‐time indicator of chemotherapeutic efficacy in patients with advanced NSCLC and related to PFS.

## 1 INTRODUCTION

Lung cancer is the leading cause of cancer‐related death in the world. It is often detected only in advanced stages, and the 5‐year survival rate is currently 18%.^1^ Annually, approximately 1.8 million of new lung cancer cases are diagnosed worldwide, and this disease contributes to 1.6 million deaths.^2^ Nonsmall cell lung cancer (NSCLC) accounts for the majority of all lung cancer cases (85%).^3^ Systemic chemotherapy is the primary treatment for advanced NSCLC, and it can extend the survival duration of patients.^4^, ^5^, ^6^, ^7^

Several studies report that the change in computed tomography (CT) is indicative of monitoring chemotherapy response.^8^, ^9^, ^10^ However, it cannot be used for monitoring timely response to chemotherapy and prediction of progression‐free survival (PFS). In addition, the requirement for specialized equipment, high cost, and potential damage to the patient’s body limit the application of CT scanning. Seeking effective and efficient new tumor biomarkers for monitoring response to chemotherapy has been a major focus in the field of clinical cancer research.

In our previous study, a monoclonal antibody (McAb), designated NJ001, which could react to NSCLC cells, was found from home‐made cancer monoclonal antibody library. The corresponding antigen of NJ001, SP70, a protein with the relative molecular mass (Mr) of 70 kDa, was demonstrated to be located in the cytoplasm and on NSCLC cells membrane. NJ001 could effectively inhibit SPC‐A1 cell proliferation in vitro and in vivo by blocking cell membrane SP70 and induce apoptosis in SPC‐A1 cells.^11^ Also, our recent study showed that higher SP70 level can be detected in the serum and pleural effusion of patients with NSCLC. Nevertheless, the relationship between serum SP70 levels and the efficacy of different chemotherapeutic agents has not been investigated. The aim of this study was to assess the role of SP70 on predicting chemotherapeutic response and PFS for patients with NSCLC.

## 2 MATERIALS AND METHODS

### 2.1 Study population

A total of 152 patients with stage III and IV NSCLC were recruited in this retrospective cohort study from the First Affiliated Hospital of Nanjing Medical University from 2015 to 2017. The inclusion criteria were (1) histologically or cytologically confirmed NSCLC, (2) newly diagnosed disease, (3) inoperable or metastatic disease, (4) at least 1 measurable carcinoma lesion, (5) an Eastern Cooperative Oncology Group (ECOG) performance status (PS) from 0 to 2, and (6) recipients of chemotherapy regimens for at least 2 cycles. The following data were collected from each patient’s medical records, including age, gender, smoking status, date of initiation of chemotherapy, and date of disease progression during or after chemotherapy. All patients were followed up till November 2017.

This study was approved by the Ethics Committee of The First Affiliated Hospital of Nanjing Medical University (Nanjing, China), and informed consent was obtained from all participants.

### 2.2 Evaluation of chemotherapeutic efficacy

After every 2 cycles of chemotherapy, tumor response was assessed by CT scanning using the Response Evaluation Criteria in Solid Tumors (RECIST 1.1) guidelines.^12^ Responses to chemotherapy were classified as follows, including complete remission (CR), partial remission (PR), stable disease (SD), and progressive disease (PD). PFS refers to the time from the date of first treatment to the date of progressive disease confirmed by imaging or last follow‐up.

### 2.3 Serum tumor marker detection

Blood samples were collected from 152 consecutive patients prior to and after the second chemotherapy cycle. Specimens were centrifuged at 3000 rpm for 10 minutes at room temperature, and then the serum was isolated. All blood samples were processed within 2 hours of collection.

Serum SP70 was detected with sandwich ELISA kit (Code Biotech, Jiangsu, China) using polyclonal antibody (PcAb) for SP70 antigen capture, McAb NJ001 and a HRP‐labeled secondary antibody (HRP‐IgG). The levels of carcinoembryonic antigen (CEA), cytokeratin 19 fragment (CYFRA21‐1), and neuron‐specific enolase (NSE) were measured by electrochemiluminescence immunoassay (ECLIA) on a Roche cobas e602 modular analyzer (Roche Diagnostics, Basel, Switzerland).

Serum levels of SP70, CEA, CYFRA21‐1, and NSE were considered elevated when the values were equal or greater than 7.5 ng/mL, 4.7 ng/mL, 3.3 ng/mL, and 16.3 ng/mL, respectively.

### 2.4 Assessment of *EGFR* mutations and SP70 expression in tissues

Eighty‐two paraffin‐embedded tumor tissues from patients with NSCLC were analyzed. All specimens were obtained from the original biopsy before chemotherapy. DNA was extracted from paraffin‐embedded tissue sections, and the *EGFR* gene mutation status was determined using *EGFR* mutation detection kit (Amoy Diagnostics, Xiamen, China) according to the manufacturer’s instructions. Meanwhile, paraffin‐embedded tissues were processed into 4‐μm‐thick sections, SP70 expression was detected with immunohistochemistry (IHC) kit (Code Biotech, Jiangsu, China). All the specimens were examined by 2 investigators without communication.

### 2.5 Statistical analyses

All analyses were performed using SPSS software, version 19.0 (SPSS Inc, Chicago, IL, USA). The chi‐square test and Fisher’s exact test were used for comparison of patient characteristics group. Kruskal‐Wallis and Wilcoxon matched‐pairs signed‐rank test were used for nonparametric comparison among groups with different chemotherapy responses. ROC curves were constructed to compare the performance of tumor marker in predicting chemotherapy response. Kaplan‐Meier analysis with the log‐rank test was used for assessment of PFS rate. Results were considered statistically significant at two‐sided *P* values <.05.

## 3 RESULTS

### 3.1 Patient characteristics

The characteristics of patients with advanced NSCLC are summarized in Table 1. Of 152 patients recruited, 96 patients (63%) were male, and 91 (60%) were never smokers. The median age was 60 years (range 32‐87 years). Adenocarcinoma was the most common subtype. Thirty‐seven patients were classified as stage III and 115 patients with stage IV. Baseline levels of serum SP70 were elevated in ninety patients (59.2%). Besides, positive rates of CEA, CYFRA21‐1, and NSE (56.6%, 42.1%, and 34.9%, respectively) were significantly lower than SP70 in NSCLC (χ^2^ = 24.736, *P *<* *.001). Notably, patients with lymph node metastasis had higher serum SP70 levels than patients who did not have lymph node metastasis (8.27 ng/mL vs 6.56 ng/mL, *P *=* *.008). However, no significant association was observed between serum SP70 levels and age, gender, smoking history, or histological type.

**Table 1:** Clinical characteristics and serum SP70 levels before chemotherapy

Clinical characteristics	n (%)	SP70 (ng/mL)^a^	*P* value
Age (y)			
<60	73 (48%)	7.20 (5.98‐12.13)	.117
≥60	79 (52%)	8.48 (6.68‐10.50)	
Sex			
Male	96 (63%)	8.27 (6.74‐11.85)	.149
Female	56 (37%)	7.69 (5.98‐9.68)	
Smoking history			
Smoker	61 (40%)	8.71 (6.46‐13.33)	.104
Never smoked	91 (60%)	7.71 (6.44‐9.76)	
Tumor stage			
III	37 (24%)	8.00 (5.92‐9.96)	.589
IV	115 (76%)	8.15 (6.45‐11.95)	
Tumor type			
Adenocarcinoma	125 (82%)	8.00 (6.06‐10.20)	.188
Squamous carcinoma	27 (18%)	8.48 (7.01‐13.86)	
Lymph node metastasis			
Yes	126 (83%)	8.27 (6.79‐11.91)	.008
No	26 (17%)	6.56 (4.56‐8.89)	

a SP70 levels are presented as median (IQR range).

After 2 cycles of chemotherapy, the 152 patients were assessed, 30 patients achieved PR, 86 patients had SD, and 36 patients had PD.

### 3.2 Tumor biomarker SP70 and the others

**Figure 1:**
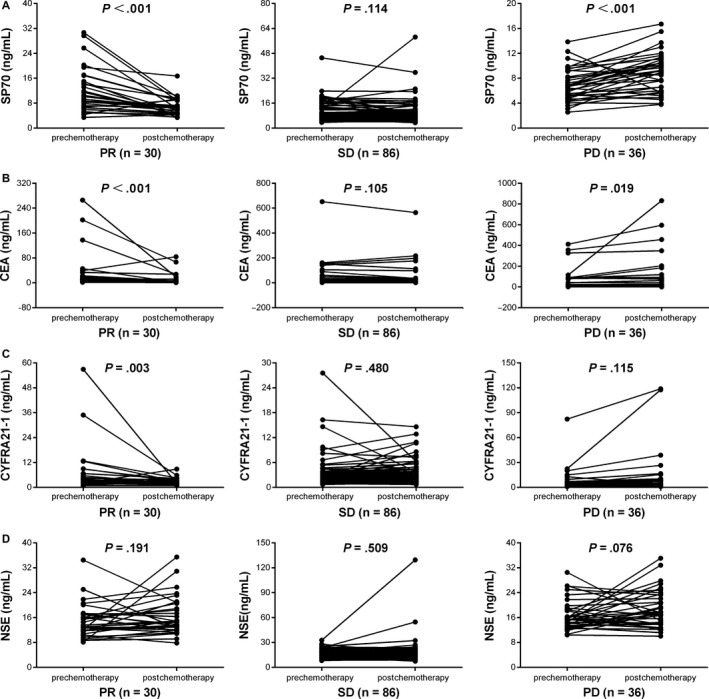
Tumor marker levels before and after 2 cycles of chemotherapy in patients grouped by different chemotherapy responses. A, SP70. B, CEA. C, CYFRA21‐1. D, NSE

Serum SP70 levels were markedly decreased in patients with PR after 2 cycles of chemotherapy (10.30 ng/mL vs 6.60 ng/mL, *P *<* *.001; Figure 1A). In contrast, no significant differences were observed in patients with SD (8.14 ng/mL vs 7.90 ng/mL, *P *=* *.114; Figure 1A). The median SP70 level in 36 patients with PD increased from 6.70 ng/mL to 9.22 ng/mL (*P *<* *.001). SP70 level decrease rates in patients with PR and SD were 31.31% (95% CI, 15.30‐44.10) and 2.67% (95% CI, ‐12.14‐9.21), respectively, whereas SP70 level increase rate in patients with PD was 28.99% (95% CI, 21.48‐51.86) within 2 cycles of chemotherapy (Table 2).

**Table 2:** SP70, CEA, CYFRA21‐1, and NSE levels and responsive rates

Tumor markers	PR	SD	PD
SP70			
Median baseline SP70 level, ng/mL	10.30	8.14	6.70
Median postchemotherapy SP70 level, ng/mL	6.60	7.90	9.22
Median difference, %	31.31	2.67	−28.99
Reduced compared with baseline, n	26	49	6
Elevated compared with baseline, n	4	37	30
CEA			
Median baseline CEA level, ng/mL	7.57	4.86	12.24
Median postchemotherapy CEA level, ng/mL	4.29	4.24	14.74
Median difference, %	25.49	5.51	−7.25
Reduced compared with baseline, n	25	49	15
Elevated compared with baseline, n	5	37	21
CYFRA21‐1			
Median baseline CYFRA21‐1 level, ng/mL	3.54	2.54	3.57
Median postchemotherapy CYFRA21‐1 level, ng/mL	2.31	2.30	3.19
Median difference, %	31.32	9.52	−26.06
Reduced compared with baseline, n	21	48	15
Elevated compared with baseline, n	9	38	21
NSE			
Median baseline NSE, ng/mL	13.31	14.19	15.42
Median postchemotherapy NSE level, ng/mL	14.67	14.28	17.74
Median difference, %	−10.63	−0.34	−5.99
Reduced compared with baseline, n	10	43	18
Elevated compared with baseline, n	20	43	18

CEA, carcinoembryonic antigen; NSE, neuron‐specific enolase; CYFRA21‐1, cytokeratin 19 fragment; PR, partial remission; PD, progressive disease; SD, stable disease.

A significant reduction in CEA and CYFRA21‐1 levels was observed in patients with PR after 2 cycles of chemotherapy (*P *<* *.001 and *P *=* *.003, respectively). For patients with PD, CEA levels increased significantly compared with its baseline level (*P *=* *.019; Figure 1B). On the other hand, CYFRA21‐1 levels increased but not significantly in patients who suffered from SD and PD (*P *=* *.480 and *P *=* *.115, respectively; Figure 1C). The median CEA level changes of patients with PR, SD, and PD were 25.49%, 5.51%, and ‐7.25%, respectively. The median changes of CYFRA21‐1 level in patients with PR, SD, and PD were 31.32%, 9.52% and −26.06%, respectively (Table 2). Unfortunately, after 2 cycles of chemotherapy, all NSE levels of patients with PR, SD, and PD were increased but not significantly (*P *=* *.191, *P *=* *.509 and *P *=* *.076, respectively; Figure 1D).

After 2 cycles of chemotherapy, among PD group, there was 83.3% (30/36) with elevated SP70 level, 58.3% (21/36) with elevated CEA level, 58.3% (21/36) with elevated CYFRA21‐1 level, and 50% (18/36) with elevated NSE level (χ^2^ = 9.6, *P *=* *.022). Among 30 NSCLC patients with PR, there were 26 cases with reduced SP70 level (86.7%), 25 cases with reduced CEA level (83.3%), 21 cases with reduced CYFRA21‐1 level (70%), and 10 cases with reduced NSE level (33.3%) (χ^2^ = 24.801, *P *<* *.001).

**Figure 2:**
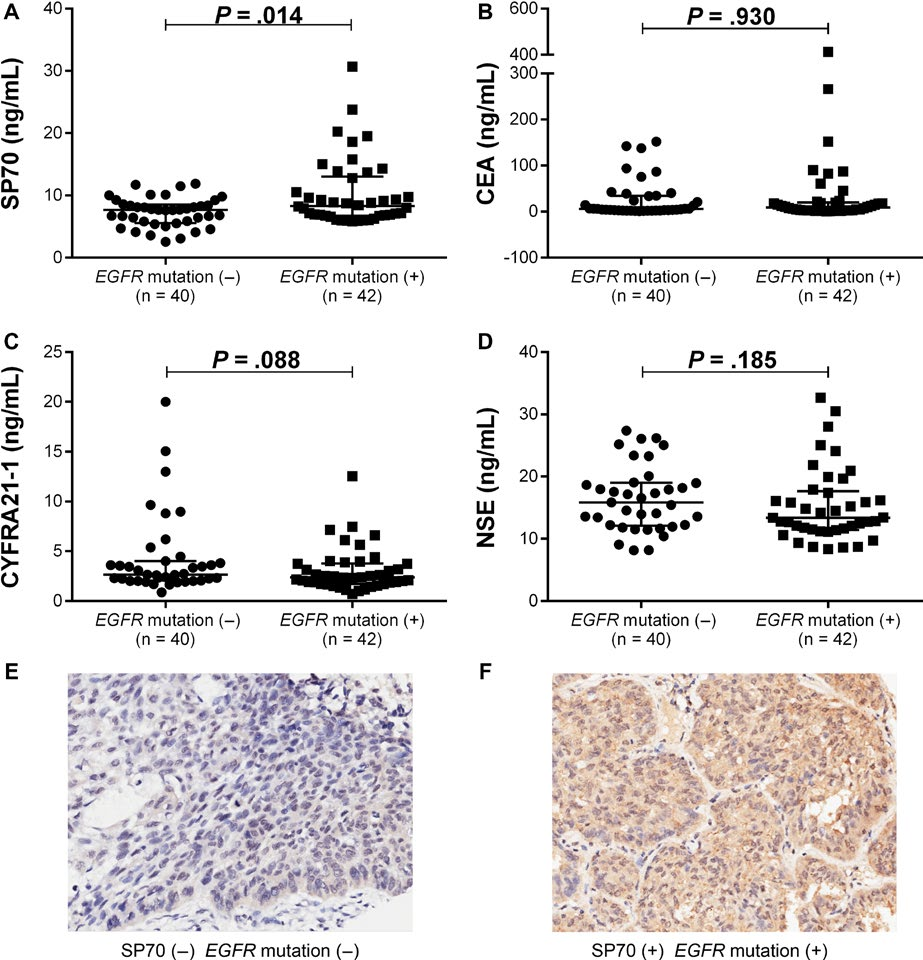
(A‐D) Tumor marker levels in NSCLC patients with different *EGFR* mutation status. A, SP70. B, CEA. C, CYFRA21‐1. D, NSE. (E‐F) SP70 expression in tissues of lung adenocarcinoma (IHC, 200×). E, SP70(−) *EGFR* mutation(−). F, SP70(+) *EGFR* mutation(+)

Overall, 82 patients with NSCLC were classified into 2 groups with available *EGFR* mutation information. As shown in Figure 2, patients with *EGFR* mutations had higher SP70 levels than patients without *EGFR* mutations (*P *=* *.014), while no correlation was seen between CEA, CYFRA21‐1, and NSE expression and *EGFR* mutations (*P *=* *.930, *P *=* *.088 and *P *=* *.185, respectively).

### 3.3 ROC analysis

**Figure 3:**
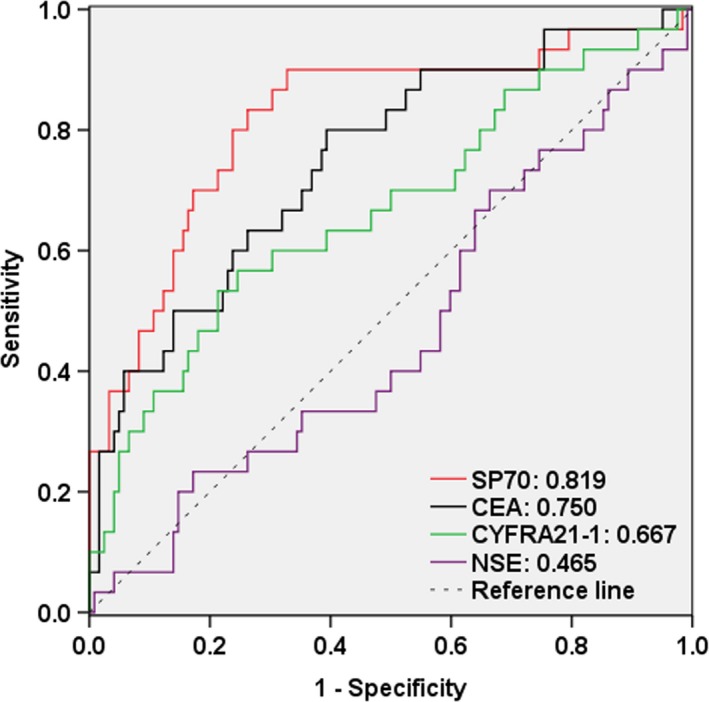
ROC curves for predicting chemotherapy responses in patients with NSCLC using reducing rates in the levels of tumor markers after 2 cycles of chemotherapy. *Red*, SP70, AUC: 0.819 (95% CI 0.725‐0.912); *black*, CEA, AUC: 0.750 (95% CI 0.650‐0.850); *green*, CYFRA21‐1, AUC: 0.667 (95% CI 0.550‐0.783); *purple*, NSE, AUC: 0.465 (95% CI 0.349‐0.582)

ROC curve analysis was performed to assess whether the decrease of these tumor markers levels can predict PR patients after chemotherapy. The areas under the curve (AUCs) for SP70, CEA, CYFRA21‐1, and NSE in patients with PR were 0.819 (95% CI, 0.725‐0.912), 0.750 (95% CI, 0.650‐0.850), 0.667 (95% CI, 0.550‐0.783), and 0.465 (95% CI, 0.349‐0.582), respectively (Figure 3).

### 3.4 *EGFR* mutation and SP70 expression in tissues

SP70 expression was detected in the cytomembrane and cytoplasm of the tumor cells. Of the 82 patients with NSCLC, 14 cases were categorized as negative (Figure 2E), 68 cases were positive staining (Figure 2F). Among these patients, there were forty cases without *EGFR* mutations. While the other 42 cases with *EGFR* mutations were distributed as follows, 1 patient had exon 18 mutation, 20 patients had exon 19 deletion mutation, 4 patients had T790M mutation in exon 20, 14 patients had exon 21 mutation, 1 patient had both exon 18 and exon 21 mutations, and 2 patients had both exon 20 and exon 21 mutations. Patients with *EGFR* mutations had higher SP70 expressions in tissue than those without EGFR mutations (χ^2^ = 9.217, *P *=* *.002).

### 3.5 Survival

**Figure 4:**
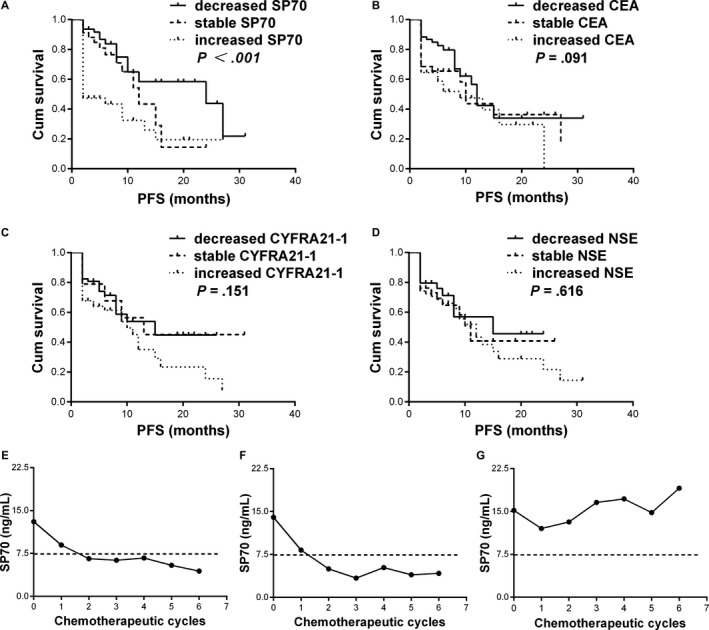
(A‐D) Kaplan―Meier curves for PFS in patients with or without reduced levels of serum tumor markers. A, SP70. B, CEA. C, CYFRA21‐1. D, NSE. (E‐G) The SP70 dynamic changes of 3 representative patients from the entire cohort during chemotherapy. Cum, cumulative; PFS, progression‐free survival

The median follow‐up duration was 7 months. Based on the changes of tumor markers levels less or greater than 10% after 2 cycles of chemotherapy, 152 patients were divided into 3 groups, including patients with decreased levels, patients with stable levels, and patients with increased levels. The median PFS for patients with decreased SP70 levels after 2 cycles of chemotherapy was 24 months, compared with 12 months for patients with stable SP70 levels and 2 months for patients with increased SP70 levels (*P *<* *.001; Figure 4A). For patients with decreased, stable, and increased CEA levels, the median PFS was 12, 10, and 9, respectively (*P *=* *.091; Figure 4B). Besides, similar results were shown in CYFRA21‐1 (15 months vs 13 months vs 10 months, *P *=* *.151; Figure 4C) and NSE (15 months vs 11 months vs 12 months, *P *=* *.616; Figure 4D).

### 3.6 Kinetics of serum SP70 levels during chemotherapy

Three representative patients were chosen for the investigation of dynamic changes in SP70 levels. All 3 patients received 6 cycles of first‐line chemotherapy. CT scanning showed significant tumor shrinkage and decreased SP70 levels in patient 1 and patient 2. Until the last available follow‐up date, these 2 patients were still alive (21 months and 22 months; Figure 4E,F). In contrast, the condition of patient 3 deteriorated due to brain metastasis. This patient’s serum SP70 levels showed a sustained increase, and the patient died 12 months after diagnosis (Figure 4G).

## 4 DISCUSSION

Due to the variable responses of patients to chemotherapy, an effective monitoring method for assessing response during the early phase of treatment is essential. Imaging detection remains the major method to evaluate responses to chemotherapy.^13^ While, sometimes, it is difficult to accurately and timely assess changes of target lesions through imaging, which makes it difficult to interpret true responses to chemotherapy, especially at the early stage of the treatment.^14^, ^15^, ^16^ Numerous studies have shown that if chemotherapy is effective, the serum levels of several tumor biomarkers decrease compared with the levels before treatment. Whereas the levels can significantly increase again during tumor recurrence, suggesting that serum tumor biomarkers can be used to evaluate treatment efficacy and predict tumor recurrence.^17^, ^18^ Recently, the availability of companion biomarkers could improve drug efficacy, decrease toxicity, and lead to a more individualized approach to cancer treatment. Anaplastic lymphoma receptor tyrosine kinase (*ALK*) translocations are used for identifying patients with NSCLC likely to benefit from crizotinib.^19^, ^20^ However, existing biomarkers are not sensitive enough to provide real‐time information on the effectiveness of chemotherapy.

Anticancer drugs can induce cancer cell apoptosis.^21^, ^22^, ^23^ The apoptotic dynamic change of cancer cell is complicated, and image of the tumor lesion is varied, especially at the early stage of the treatment.^24^, ^25^ SP70 was particularly expressed in lung cancer cells, and it has been shown to reflect tumor mass by correlating with tumor proliferation and apoptosis.^11^ As the half‐life time of SP70 is about 3 days, serum SP70 levels should decrease rapidly after effective therapy due to successful clearance of tumor cells.

We demonstrate that serum SP70 levels are highly increased in patients with NSCLC and are decreased in patients with the effective chemotherapy. Serum SP70 showed highest sensitivity in predicting the chemotherapy responses in the early stage of treatment compared to some other tumor markers available.

In recent years, several studies have reported that the level of serum tumor biomarkers is closely related to the diagnosis and treatment of lung cancer and can be used for assessing the effectiveness of chemotherapy or radiotherapy.^26^, ^27^, ^28^ We simultaneously detected and conducted comparative analysis of the tumor markers CEA, CYFRA21‐1, and NSE. After 2 cycles of chemotherapy, the levels of CEA and CYFRA21‐1 were increased in patients who experienced PD and were significantly decreased in patients with PR. However, we found no significant changes in NSE level in the overall cohort. One possible explanation for this discrepancy is that we recruited patients with NSCLC in our cohort, while NSE is primarily a marker for the diagnosis and monitoring of treatment in patients with small cell lung cancer (SCLC).^29^, ^30^ In this study, the changes of SP70 levels are obviously greater than above tumor biomarkers. Furthermore, an ROC curve analysis for predicting PR to chemotherapy indicates that SP70 was superior to other biomarkers. These results suggested that SP70 is more sensitive and closely correlated with response to chemotherapy in patients with advanced NSCLC compared to the present tumor markers in use.

Moreover, the median PFS for patients with decreased SP70 levels after chemotherapy is longer than that of patients with increased SP70 levels. The results of Kaplan‐Meier analysis and dynamic changes in SP70 levels indicate that patients with increased SP70 levels after treatment have shorter PFS than patients with stable or decreased SP70 levels. Therefore, an increase of serum SP70 level is the indication of poor survival. In clinical application, serum tumor biomarkers can be used as prognostic indicators in NSCLC.^31^, ^32^ Chiu et al^33^ revealed that changes of CEA, CA125, and CA19‐9 levels at 4 weeks after treatment with gefitinib can predict the survival of patients with advanced NSCLC, and unexpectedly, the authors found no correlation between CEA and OS or PFS. Ma et al^34^, ^35^ investigated the predictive value of CYFRA21‐1 and other serum biomarkers in patients with NSCLC and identified that CYFRA21‐1 was an independent prognostic factor for OS. However, other studies demonstrated that CEA level was not a prognostic factor in populations with a higher burden of smoking‐induced lung carcinogenesis and had no association with PFS or OS.^36^, ^37^ In our study, Kaplan‐Meier analysis demonstrates no significant difference of survival time between patients with increased, stable, or decreased CEA, CYFRA21‐1, and NSE levels. These results indicate that SP70 is more significant than other tumor markers in predicting PFS.

Subsequently, we found that patients with *EGFR* mutations had higher serum SP70 levels and higher tissue SP70 expression than patients without *EGFR* mutations. The mechanism underlying the positive correlation between SP70 expression and *EGFR* mutation is unclear. Our previous study showed that SP70 is a tumor key protein which can regulate numerous gene expression (GEO accession number: GSE59655) in cancer cells promoting cancer cell proliferation and metastasis, and this may be related to the gene mutation of *EGFR*. Additionally, monoclonal antibody NJ001 can induce NSCLC tumor cell apoptosis. Although several studies reported that *EGFR* mutations are associated with elevated levels of serum CEA, especially in patients with lung adenocarcinoma,^38^, ^39^ no correlation was found between CEA concentration and *EGFR* mutation in this present study.

Our previous study indicates that tumor suppressor gene promoter methylations are meaningful biomarkers for short‐term chemotherapy response assessment.^40^ While sensitive, real‐time methylation‐specific PCR (MSP), and some other techniques for DNA methylation detection are comparatively complicated and time‐consuming, their clinical applications may be limited.

This is the first study on SP70 as a monitoring biomarker for the response to chemotherapy in patients with advanced NSCLC. However, follow‐up duration can be prolonged in our future study to evaluate the association between serum SP70 and overall survival. As for advanced patients with NSCLC who do not have obvious changes of imaging and routine serum tumor biomarker, the detection of SP70 level is of great clinical significance. Measurement of serum SP70 levels is objective compared to CT scanning, clinically available, and easily obtained.

In conclusion, this study demonstrates that serum SP70 level detection is a more sensitive indicator to predict timely response to chemotherapy and PFS.

## CONFLICT OF INTEREST

We declare that no competing of interest and conflicts of ethics involved in the manuscript.
